# A decentralized hybrid computing consumer authentication framework for a reliable drone delivery as a service

**DOI:** 10.1371/journal.pone.0250737

**Published:** 2021-04-30

**Authors:** Abdul Hannan, Faisal Hussain, Noman Ali, Muhammad Ehatisham-Ul-Haq, Muhammad Usman Ashraf, Ahmed Mohammad Alghamdi, Ahmed Saeed Alfakeeh

**Affiliations:** 1 University of Management and Technology, Sialkot, Pakistan; 2 Al-Khwarizmi Institute of Computer Science (KICS), University of Engineering and Technology (UET), Lahore, Pakistan; 3 Sino-Pak Center for Artificial Intelligence (SPCAI), Pak-Austria Fachhochschule: Institute of Applied Sciences and Technology, Haripur, Khyber Pakthunkhwa, Pakistan; 4 Department of Software Engineering, College of Computer Science and Engineering, University of Jeddah, Jeddah, Saudi Arabia; 5 Department of Information Systems, Faculty of Computing and Information Technology, King Abdulaziz University, Jeddah, Saudi Arabia; Xidian University, CHINA

## Abstract

The thriving adoption of drones for delivering parcels, packages, medicines, etc., is surging with time. The application of drones for delivery services results in faster delivery, fuel-saving, and less energy consumption. Giant companies like Google, Amazon, Facebook, etc., are actively working on developing, testing, and improving drone-based delivery systems. So far, a lot of work has been done for improving the design, speed, operating range, security of the delivery drones, etc. However, very limited work has been done to ensure a complete and reliable last-mile delivery from the merchant’s store to the hands of the actual customer. To ensure a complete and reliable last-mile delivery, a drone must authenticate the consumer before dropping the package. Therefore, in this work, we propose a consumer authentication (Consumer-Auth) hybrid computing framework for drone delivery as a service to make sure that the parcel is perfectly delivered to the intended customer. The proposed Consumer-Auth framework enables a drone to reach the exact destination by using the GPS coordinates of the customer autonomously. After reaching the exact location, the drone waits for the customer to come to the specific pinned location then it starts a two-factor consumer authentication process, i.e., one-time password (OTP) verification and face Recognition. The experimental results manifest the effectiveness of the proposed Consumer-Auth framework to ensure a complete and reliable drone-based last-mile delivery.

## 1 Introduction

A drone or unmanned aerial vehicle (UAV), is an aircraft that flies autonomously with no onboard pilot. With the advancement in technology, UAVs have gained rapid improvements in design, shape, size, communication range, and applications [1, 2]. Nowadays, drones are being used in military, civilian, agriculture, and various other sectors for different purposes like security, environment monitoring, surveillance, shipping goods, etc., due to their flexibility, versatility, easy installation, low operation, and maintenance cost [1].

Generally, a drone is a flying machine that consists of multiple sensors, rechargeable batteries, cameras, and 3-8 rotors (also called propellers) to fly. Based upon the number of rotors, the drones are named as tri-copter, quad-copter, hexacopter, and octocopter which consists of three, four, six, and eight rotors respectively [2]. Likewise, based upon the weight, payload carriage capacity, operating area range, and altitude, the drones are classified into five categories, i.e., Nano, Micro, Mini, Small, and Tactical drones [2]. These characteristics of each category are defined in Table 1. Currently, the Nano, Mini, and Microdrones are used for civilian purposes while the Small and Tactic drones are used for military purposes [2]. Based upon the wing design, a drone is classified into two types, i.e., fixed-wing drones and rotary-wing drones [3]. The fixed-wing drones have long-distance coverage along with high payload capacity as compared to the rotor-wing drones. On the other hand, the rotor-wing drones have the ability to land and take-off vertically and hover during the flight. Thereupon, the rotor-wing drones are useful for delivering parcels in urban areas situations due to their short-range, vertical land and take-off functionalities whereas the fixed-wing drones are useful for delivering parcels in rural areas due to their long area coverage capacity [3].

**Table 1:** Classification of drones [2].

Category	Weight	Operating Altitude	Operating Range	Payload Capacity
Nano	<0.2 Kg	<90 m	90 m	<0.2 Kg
Micro	0.25-2 Kg	<90	5 Km	0.2-0.5 Kg
Mini	2-20 Kg	<900 m	25 Km	0.5-10 Kg
Small	<150 Kg	<1500 m	50-100 Km	5-50 Kg
Tactical	>150 Kg	<3000 m	>200 Km	25-200 Kg

The architecture of a UAV system consists of three main units which include a flight controller, ground control station, and communication data-link [4]. The flight controller is embedded on the aircraft; it is the central processing unit of the aircraft. While the ground control station helps the human operators to monitor and control the aircraft operations during its flight. The purpose of a communication data link is to transmit the information from the ground control station to aircraft and vice-versa.

The evolution of drone technology is moving towards providing an alternative for goods transportation [2]. The drones are now being used for delivering medicine, food, parcel, packages, and other goods. The application of drones for delivery services results in faster delivery, save fuel, and energy consumption. Although the services provided by the drones are convenient, fast, cost-effective, energy-saving, and attractive, their reliance on cyber-capabilities has great security and privacy concerns. Nowadays, the adoption of drones for fast and cost-effective parcel delivery is escalating. The giant companies like Google, Amazon, Facebook, DHL, etc., are actively working on developing, testing, and improving drone-based delivery systems to circumvent the security, privacy, and efficiency constraints [5].

So far, a lot of work has been done for improving the design, speed, operating range, security protocols for drones, operating regulations for drones, privacy and safety aspects of the delivery drones, etc. However, very limited work has been done to ensure a complete delivery from the merchant store to the hands of the actual customer. In short, the current drone delivery systems lack consumer authentication. Therefore, in this work, we raise attention about the customer authentication aspect for ensuring the correct delivery of the parcel to the intended customer.

In conventional logistics, goods transport, last-mile delivery is a challenging task [6]. There exist some insecure areas, where conventional goods transport refuses to deliver the parcel. Similarly, in the flood era, or some other natural disaster situation, last-mile delivery using conventional means becomes impossible. However, a drone can be used for parcel delivery in all such situations [7]. During the existing COVID-19 breakout, the drone delivery systems have achieved much attention and significance due to their potential to deliver the parcels faster without doing any physical contact with human beings [8, 9]. Moreover, to impede the fear of people in visiting COVID-19 testing facilities, the drones can be used to collect and bring the test samples of the population into the laboratory [9].

The existing drone delivery systems deliver the parcel at a centralized drop-off location [10]. Although, Amazon, Google, UPS, and many other parcel delivery drones have very impressive videos showing the autonomous delivery of parcels via drones yet their drones deliver parcels to a centralized drop-off location either in rural areas or in an urban area with no other house around [10, 11]. The autonomous last-mile delivery becomes a crucial challenge when deploying it in urban areas [10]. This is due to the proliferation of multi-story buildings, residential societies, apartments, etc., in urban environments [12]. In such an urban environment, besides finding the right building, the drone needs to reach the exact apartment is to complete the last mile delivery [10]. Furthermore, the identification of the exact destination is not merely enough since there exist some cases in which the parcel is not delivered to the customer. For example, if a drone simply drops the package in front of a customer’s doorstep but at that time the customer is not present at home, and the package is stolen from the customer’s doorstep before the customer arrives to receive it. Hence, for making complete last-mile delivery, besides the identification of the exact destination for landing, a drone must also authenticate the customer to prevent repudiation [11]. Therefore, in this work, we propose a decentralized hybrid computing consumer authentication (Consumer-Auth) framework for a reliable and secure drone delivery system to make sure that the parcel is correctly delivered to the intended customer.

The proposed Consumer-Auth framework enables a drone to reach the exact destination by using the GPS coordinates of the customer autonomously. After reaching the exact location, the drone waits for the customer to come to the specific pinned location then it starts a two-factor consumer authentication process, i.e., face recognition and one-time password (OTP) verification. Moreover, the above-mentioned consumer verification process is used to identify the recipient with the adoption of a proposed hybrid computing mechanism to minimize latency and energy consumption. In case, if a consumer is not recognized or does not enter the correct OTP, the drone waits for some time and again start authenticating the recipient. In the worst-case scenario, if a recipient did not appear at the required location or did not recognize as the actual recipient, the drone returns to the initial location without delivering the package.

### 1.1 Research contributions

The main contributions of this research work are as follows:

1. We proposed a consumer authentication (Consumer-Auth) framework to ensure a complete and reliable last-mile delivery using drones.
2. We used hybrid computing to minimize the response time of consumer authentication via the two-factor verification method.
3. We designed a customized drone prototype to implement and test the efficiency of the proposed Consumer-Auth framework in a real-time Drone Delivery as a Service.
4. We evaluated and analyzed the performance of the proposed framework by comparing the existing cloud and edge computing mechanisms with the proposed hybrid computing architecture on the Consumer-Auth framework.

The rest of the paper is organized as follows: Section 2 presents a review of some existing work for the drone as a delivery system. Section 3 describes the proposed consumer authentication framework for reliable last-mile delivery. Section 4 explains the communication flows of the proposed system architecture for a reliable consumer authentication based drone delivery as a system. Section 5 describes the experimental setup and the performance parameters used in this study. Section 6 discusses the results of the proposed framework. Lastly, Section 7 concludes the paper.

## 2 Literature review

The adoption of drones as a delivery system has become an attractive area of research and development nowadays. Many giant companies like Google, Amazon, etc., are actively working on developing, testing, and improving drones delivering goods. Recently, Amazon started using a drone delivery system for 86% of packages weighing up to 5 pounds [13]. Likewise, the United States Postal Service (USPS) is also working on using drones over the traditional delivery systems to deliver packages due to the rapidness and cost-effectiveness of the system [14]. The USPS strategy would particularly tackle the rural areas where the distribution of goods to the households is unlikely difficult.

Several studies have been conducted to optimize the design, operating area range, payload carriage capacity, delivery time, battery time, security, privacy, flight legislation, etc., of drones to pledge the reliable applications of drones in our daily life. Babar *et al*. [15] proposed a framework to minimize the drone delivery time and operation cost. The authors followed a Spatio-temporal service model and a quality model and proposed a heuristic approach to reduce both the time and cost of a drone delivery system. Li *et al*. [16] depicted the delivery of weights using drones in a three-dimensional (3D) environment which gets its coordinates set from takeoff position to the destination via mobile application. The presented module is capable of flying to the supplier and gets the weight linked up then departures to the client’s spot. It integrates route optimization techniques and visual models to make the drone efficient to deliver orders in strangled and confusing areas such as homes or offices. The drone flies to the destination location and scans the QR code to land safely. A few research studies used drones for object detection and human tracking to ease the police and military operations like surveillance, tracking suspects, finding the missing persons, etc. Adam *et al*. [17] proposed a deep learning approach for drone-based facial recognition tasks. The authors compared three different deep learning algorithms out of which InceptionResNetV2 exceptionally outperformed other models. Van *et al*. [18] utilized drones for tracking ground objects. The authors proposed an approach to identify the people based upon the drone view and the wearable device data.

So far, a lot of work has been done to ensure the security and privacy of a drone’s identity and location. Geumhwan *et al*. [19] proposed an authentication framework to verify the identities of flying UAVs to prevent unauthorized drones. Their proposed framework generates a flight session key for UAVs and stores this key in a central database along with the flight plan. The ground stations can access this database to verify a drone session key and its flight plan to ensure mutual authentication between the flying UAVs and ground stations. Lin *et al*. [20] proposed a solution to secure the identity and privacy of drones flight information. Furthermore, they also discussed securing the data sharing between drones and clouds. Likewise, Ali *et al*. [21] worked on securing the surveillance data communicated through drones.

In [22], Jaihyun *et al*. proposed a modular approach for the drone delivery system instead of a primitive non-modular design. Moreover, the author described the benefits of using modular UAVs during fleet which are quite expressive and fruitful. However, during drone flight, replacement of drone modular components such as a battery, etc. is going to be challenging and it also needs well-equipped drone service stations on the path which can increase the security concerns and overall propagation delay. So, to enhance the performance of the drone delivery system, the use of a modular approach instead of built-in memory and power components is not enough. We need a proper system that utilizes minimal drone energy resources instead of component replacement during the drone fleet. By considering the above loopholes, we proposed a decentralized computations-based drone delivery system. It is quite effective due to task distribution on multiple computing models which can reduce the overall energy requirement of a drone in a UAV-based delivery system.

Moreover in [23], the author presented a fast, reliable, and cost-effective last-mile drone delivery system based on IoT technology. Moreover, in this paper, the author makes use of Long Range Wide Area (LoRA) technology with the existing geo-positioning system to improve the end to end parcel delivery mechanism. To enhance the existing drone delivery system, the author only focused on near-by optimal pathfinding which is not sufficient. There are plenty of other parameters that need to be considered to reduce the last-mile drone delivery cost and make it more reliable. To overcome the above-mentioned issue, we proposed a decentralized energy computation-based drone delivery model which does not only reduce the cost but it’s also become more secure and reliable due to the adoption of a hybrid computing mechanism.

Meanwhile, in [24], Hailong *et al*. proposed another drone delivery system with the assistance of public train routes. In this paper, drones are installed on the train roof, so it can launch from the train roof and return to their initial position after the parcel delivery task is completed. Despite its advantages, the major loophole in this work is the lack of a last-mile drone delivery option at the doorstep. Moreover, this system operated according to the existing train routes schedule which limits the overall scope of the drone delivery mechanism. In view of the above loophole, we proposed a decentralized drone delivery computation mechanism that is more efficient than the existing approaches.

In addition, Novella *et al*. [8], proposed a trustable decentralized drone delivery mechanism. In this paper, the author used the concept of a drone’s chain for last-mile delivery items. Moreover, instead of using a central drone hub, the researcher used multiple drones belongs to different owners to carry on the single parcel delivery through UAVs. The customer’s order moves through multiple drone pick-up points during its fleet which is quite challenging and difficult to ensure full-proof security. Furthermore, the author adopted a blockchain-based drone delivery mechanism to secure and to gain end-customer satisfaction. However, the multiple charges transferring in the drone chain scheme compromises the reliability factor in the overall drone delivery system. By keeping the above fact, we proposed a customer authentication based decentralized drone delivery mechanism which does not only optimize the drone energy requirements but also enhances customer satisfaction through a proper two-step customer validation process.

In [5], the author proposed a drone delivery authentication process based on acoustic noise fingerprinting. In this work, the author highlighted the differences in acoustic noise characteristics in motors due to manufacturing defects for drone identification. However, due to challenging real-time environmental parameters during the drone fleet, it’s not fruitful to fingerprint the particular drone based on motor SoundUAV particularly. There are plenty of other factors such as humidity, air pressure, motors speed, etc. which intercept the sound waves which affect the authentication system. Thus, we proposed a two-step decentralized customer authentication drone delivery mechanism that is more secure and reliable as compared to primitive approaches.

Brunner *et al*. [10] highlighted that the existing delivery drones rely on a central-drop off location. Therefore, these drones are unable to perform the autonomous last-mile delivery in the case of urban areas where the people live in a multi-story house environment. The authors called attention to make a delivery drone capable of delivering the package at other locations like porch or balcony in dense urban areas with multi-story house infrastructure. In this regard, they introduced the concept of a visual marker in which the customer/recipient simply needs to print a visual marker and stick it at the balcony door or wall or some other drop-off location. The drone will first navigate to the customer’s GPS location and then starts descending to find the drop-off location where the customer tagged the visual marker. Finally, the drone unloads the package where the visual marker is found. Likewise, Seo *et al*. [11] proposed a security framework that uses a white-box cryptography technique to secure the critical data of delivery drone. The authors also proposed a concept of e-receipt to authenticate the customer for the secure delivery of the package.

Although a lot of work has been done for improving the different aspects of drone-like design, operating range, security, privacy, etc. to surge the utilization of drones as a service in our daily life. However, the delivery drones still lack authenticating the customer identity to fully resolve the last mile delivery problem especially in the case of dense urban multi-story infrastructure. Henceforth, a very limited work like [10, 11] has been done to ensure a risk-free and trustworthy delivery of goods in the right hands. Therefore, in this work, we propose a framework that enables a drone to reach the exact destination by using the GPS coordinates of the customer autonomously and drop the package after the customer’s face recognition and verification via OTP. The following section provides a detailed description of the proposed methodology.

## 3 Proposed framework

**Fig 1:**
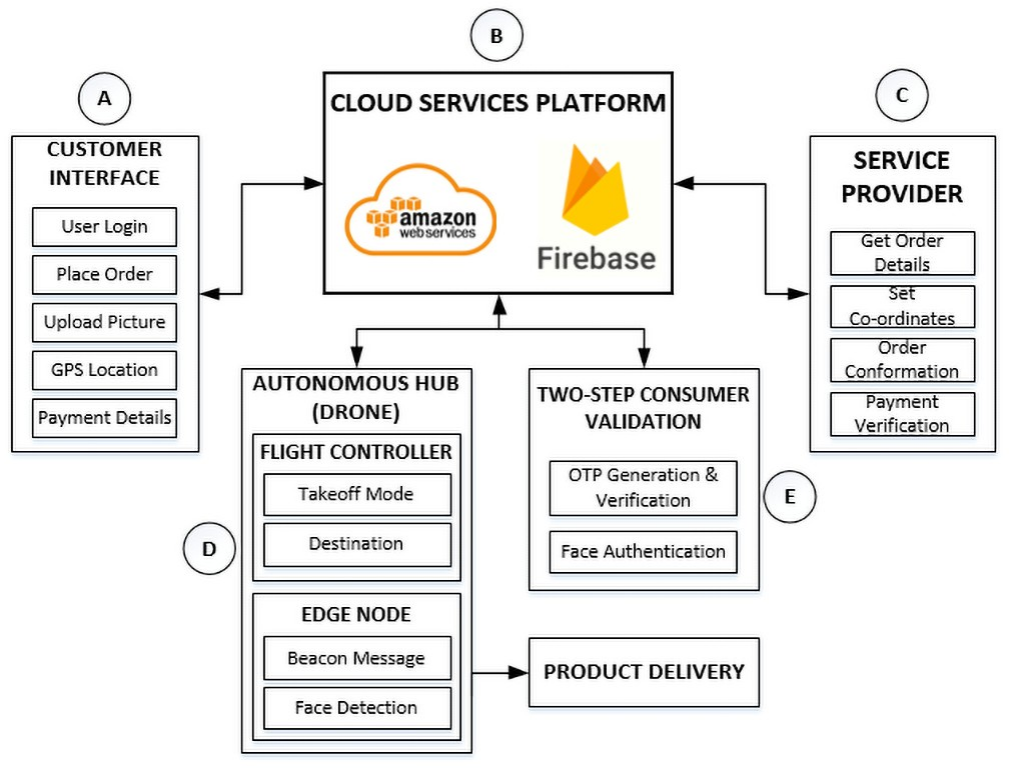
Block diagram of the proposed consumer authentication (Consumer-Auth) framework.

To ensure a complete and reliable drone-based last-mile delivery, we propose a two-factor consumer authentication (Consumer-Auth) framework. The proposed Consumer-Auth framework consists of five major modules which include customer interface, cloud platform, service provider, autonomous hub, and consumer authentication module as illustrated in Fig 1. The proposed Consumer-Auth framework triggers the user’s product order requested through the customer interface module. After that, the customer information is stored in Amazon Web service (AWS) cloud [25], where the courier service provider retrieves and approves the order and sends it to an autonomous hub (drone). Thereafter, the autonomous drone changes its flight mode from “Hold” to “Takeoff” and moves towards the customer’s destination. Using the customer’s GPS coordinates, when the drone reaches the destination, it starts executing a two-factor consumer authentication process. Finally, upon successful authentication, the product is delivered to the authentic customer. The detailed functionalities of each module are described in the following subsections.

### 3.1 Customer interface module

The customer interface is the very first module to activate the drone delivery process. This module acquires four types of information which include personal information, order information, GPS location, and payment details from a customer and then send it to the cloud services platform for order approval as shown in Fig 1. In the personal information, the customer provides the details about his/her first name, last name, address, email, contact number, and upload his/her picture. Afterward, the customer selects the desired product and place the order. After that, the customer provides the GPS location where the drone has to deliver the parcel. Finally, the customer provides information to pay for his/her order. The customer’s GPS location will be used by the drone to reach the destination. Similarly, the customer’s contact number will be used for the OTP verification authentication process while the customer’s picture will be used for face recognition done before delivering the parcel.

### 3.2 Cloud service platform

The cloud service platform is the central processing module of the proposed framework which receives, processes and transmits data to all other modules. To save time, energy, memory, and computational power on edge nodes, all the major computational tasks including data storage, OTP generation, OTP verification, and face recognition using deep learning model, are processed at cloud services platform.

The cloud services platform consists of two sub-modules which include: AWS and Firebase. The Firebase receives, processes, and stores the customer details obtained from the customer interface module. On the other hand, AWS cloud platform only executes the customer face recognition process using ‘AWS Rekognition’ module [26, 27]. It then passes this information to the service provider module where the customer’s order is approved. Besides the approval, the service provider module requests a Firebase module to share the customer’s GPS location with the drone to reach the destination. Furthermore, AWS shares the customer’s face recognition results with the Firebase which uses it for customer authentication and verification. Firebase is a realtime database that is hosted on a cloud server to centrally accessible to all connected clients [28].

### 3.3 Service provider module

The service provider module consists of an interface through which a service provider can maintain the customer’s record, order management, payment verification, and approve or reject the request. After the order confirmation, the required customer information like GPS co-ordinates is fetched from the Firebase real-time database to carry on the autonomous drone delivery flight to the given longitude and latitude coordinates. Moreover, the parcel is attached beneath the drone which will be released at the required destination after a two-step consumer verification process to identify the appropriate customer.

### 3.4 Autonomous hub (drone)

In the proposed Consumer-Auth framework, an autonomous hub (drone) is the most significant entity to accomplish the product delivery process at the exact customer’s location and deliver the parcel to the authentic customer. The autonomous hub module consists of two sub-modules which include flight controller and edge node as illustrated in Fig 1.

The flight controller module is responsible to control the takeoff and landing modes of the drone. The flight controller module takes off the drone upon receiving the instructions from the service provider via a cloud service platform. Finally, when the drone reaches the customer’s GPS location, it lands and releases the parcel after the two-step verification. On the other hand, the edge node module is responsible for capturing the customer’s image when the drone reaches a destination. After capturing the customer’s image, the edge node performs some image processing task to localize the customer’s face in the image then send it to the AWS S3 bucket storage where the ‘Amazon Rekognition’ deep learning model recognizes the authentic customer’s face concerning the image provided by the customer while placing the order. In case, if the customer’s face is not localized in a captured image, the edge node will capture another image of the customer until it localizes the customer’s face within a specified limited attempt—in our case, we limit the number of attempts to less than equal to three—and send it to the ‘Amazon Rekognition’ deep learning model for customer authentication.

### 3.5 Two-step consumer validation

This module is the key module of the proposed framework as it performs consumer authentication using a two-factor authentication mechanism. This module contains two sub-modules which include OTP verification module and face recognition module. The OTP verification module authenticates the customer by sending an OTP at customer’s mobile phone number that he/she has provided while placing the order. On the other hand, the face recognition module recognizes whether the recipient is the authenticate customer or not.

The two-step consumer validation module gets activated when the drone reaches the customer’s given location where it has to deliver the parcel. As the drone reaches a customer’s GPS location, it first starts capturing the recipient’s face image and sends it to the face recognition module. Furthermore, the customer also receives an OTP sent to the customer’s mobile phone for final authentication. The following sub-sections describe the working of these two sub-modules:

#### 3.5.1 OTP verification

This module is responsible to generate an OTP and validate the authenticity of the recipient before delivering the parcel. In this user validation process, the drone sends the beacon message to the customer’s mobile phone by using Twilio [29] API service after reaching the required destination. After waiting for the customer to come outside, a random 4-digit OTP is generated by running a python script at the edge node—a Raspberry Pi 4 (RPi-4) controller that we embedded at the drone for lightweight computation—and it is sent to the Firebase cloud service. The Firebase saves this OTP into its database and also sends it to the customer’s mobile phone.

Upon receiving the OTP, the customer enters this OTP into a mobile application which has been sent to the customer’s given contact number. The customer’s OTP verification process is done within 10 to 15 seconds approximately. After the customer’s verification, the autonomous drone changes its altitude and move downwards to a height of 4 meters away from the recipient. Finally, with a swift change in altitude and the face recognition process starts running. However, if the customer is not verified in the first attempt then the OTP is generated and sent again for verification. In the case of three attempts, if the customer is not verified via OTP then the autonomous drone returns to its initial position without delivering the parcel.

#### 3.5.2 Face recognition

**Fig 2:**
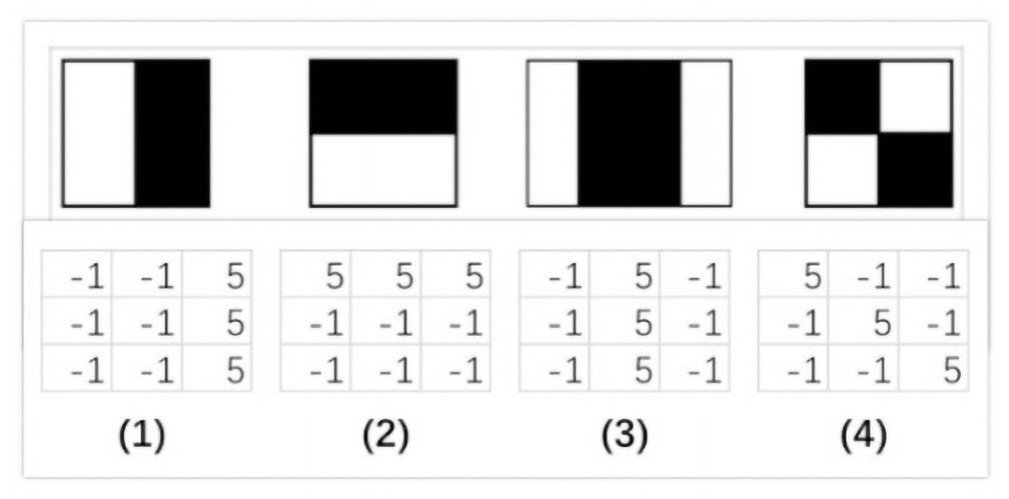
Haar algorithm features with kernel window representation.

Face recognition is the final step for the recipient’s verification. Does the face recognition module recognize whether the recipient is the authenticated customer or not? This module performs its heavy deep learning classification using hybrid computing mechanisms such as AWS cloud and edge computing. The purpose of using a hybrid computing mechanism is to get the pros of both cloud and edge computing schemes. Cloud computing is beneficial in terms of the liability of resources, but an increase in delay is the major loophole in this phenomenon. On the other hand, in edge computing, the data is computed where it is generated. Although, edge computing minimizes the delay factor, energy consumption increases which is the core concern in the drone delivery system. To circumvent these loopholes, we proposed a hybrid computing mechanism in the Consumer-Auth framework. Therefore, we first detect the recipient’s face on the edge node (RPi-4 controller) using the Haar algorithm which is deployed on the autonomous hub, i.e., drone. The Haar algorithm utilizes four major filters, i.e., line filter, rectangle filter, and two edge filters to detect a particular face. The edge features are used to detect the edges and while the line and rectangle filters are used to detect a slanted line in a given image. Fig 2, represents the visual perception of Haar features along with kernel window of each feature, i.e., (1) and (2) for edge detection, (3) for line detection, and (4) for rectangle detection which is used for face detection.

Once the recipient’s face is detected by the edge node in the captured image, this image is transmitted to the AWS cloud. At AWS cloud, we have an ‘AWS Rekognition’ service that uses a deep learning model for matching the captured image to recognize whether the recipient is the intended customer or not? Finally, after the two-step verification process is accomplished then the product is dropped at the customer’s given location and the autonomous drone fly back to its initial location.

## 4 Work flow of the proposed Consumer-Auth framework

**Fig 3:**
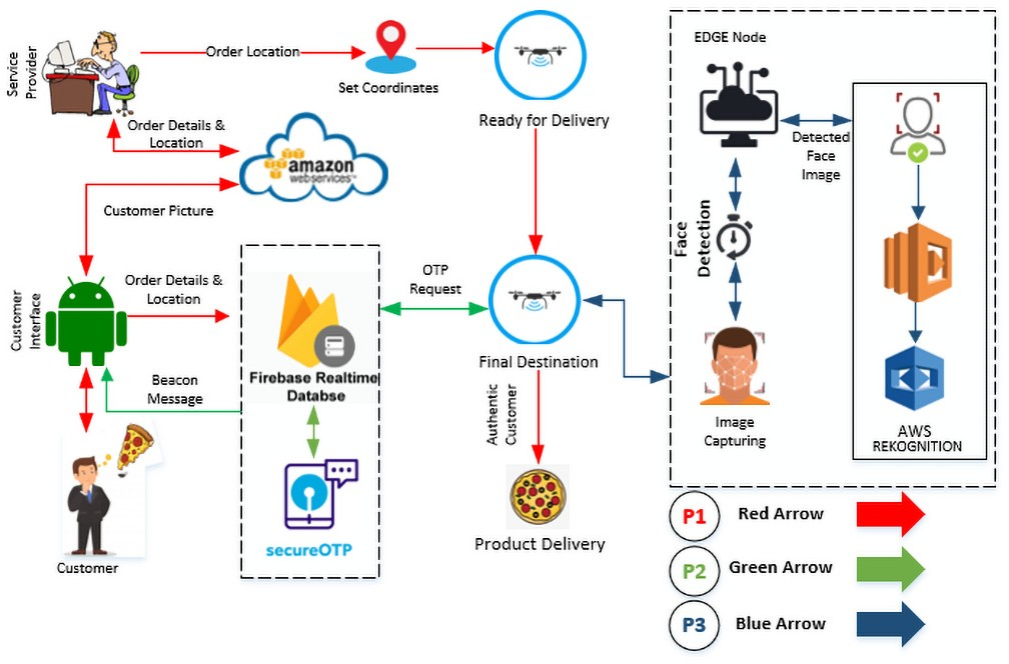
Work flow of the proposed Consumer-Auth framework.

To better illustrate the working of the proposed Consumer-Auth framework, Fig 3 displays the communication flows of the proposed system architecture for three major processes named process-1 (P1), process-2 (P2), and process-3 (P3). The P1 demonstrates the flows starting from customer order to the departure of the drone at the customer’s given destination. Similarly, the P2 illustrates flow steps for OTP generation and verification which start after the drone reaches a customer’s destination. Likewise, the P3 represents the communication flow for face recognition process which is done after the customer’s verification through OTP. In short, the drone delivery initiates with the consumer’s order, therefore, all these three processes are executed sequentially to make a reliable last-mile delivery of the product. These processes are explained in the following sub-sections.

### 4.1 From the customer’s order to the departure of drone (P1)

As discussed earlier that the proposed Consumer-Auth framework triggers the placement of order. In Fig 3, the red color arrows show the flow starting from the customer’s order to the departure of the drone at the customer’s given location. The flows with red arrows shown in Fig 3, manifests that as the customer places an order through customer interface, all the customer given details including his/her details, GPS location, product order, face image, and contact number are stored at AWS and Firebase cloud server. Afterward, a service agent of that particular courier service provider retrieves the customer’s information, verifies the payment, and approves the customer’s order for delivery through the service provider interface. Finally, the customer’s order is loaded into the autonomous hub (drone) and the customer’s GPS location and mobile number details are transferred to the drone to take off its flight automatically as shown in Fig 3.

### 4.2 OTP verification (P2)

Once the drone reaches the customer’s given GPS location, it sends a beacon message to the customer’s mobile phone to notify him/her to come to the specified location for parcel delivery, as shown in Fig 3.

Afterward, the drone generates a random 4-digit OTP and sends a request to the Firebase server for OTP verification as portrayed in Fig 3, particularly represented through green color arrows. The Firebase server first saves the received OTP into its database and sends it to the customer’s given mobile number for initializing the two-factor verification process. As the customer receives the OTP, he/she sends it to the Firebase server via customer interface for the verification as shown in Fig 3. Finally, after a successful OTP verification, the autonomous drone changes its altitude and moves downwards to a height of 4 meters away from the recipient to capture his/her image.

### 4.3 Recipient’s face recognition and product delivery (P3)

As the drone lowers its altitude and reaches a height of 4 meters away from the recipient at the given GPS location, the drone starts capturing the recipient’s picture. After capturing the recipient’s picture, the drone first detects and localizes the face of the recipient in the captured picture using the Haar algorithm by running over its edge computing module as mentioned in subsection 3.5.2. The whole process of consumer face authentication is represented through dark blue color arrows as illustrated in Fig 3. If the recipient’s face is detected in the captured picture then the drone sends it to the AWS Lambda—a module in the AWS cloud—where the AWS face Rekognition service recognizes whether the recipient is the actual customer or not through a deep learning model. In case, if the face is not detected by the edge computing module in the captured image, the drone captures another image until it detects the face in the captured image within a specified time. Similarly, if the recipient is not recognized in the first captured picture then at max two more attempts will be made to recognize the intended customer for reliable delivery. Finally, after a successful two-factor consumer authentication process, the requested product is delivered to the recipient.

In a nutshell, the proposed two-factor consumer authentication mechanism, the proposed Consumer-Auth framework enables a drone for reliable delivery of the parcel to the actual intended customer. Furthermore, the proposed Consumer-Auth framework also reduces the delay factor with the help of hybrid computing by performing the light processing task like face detection at edge node and the heavy processing tasks like face recognition using a deep learning model at the cloud edge.

**Algorithm 1 Proposed Consumer-Auth Framework**.

**Input**: DES. Co-ordinates, Customer-image, Contact No.

**Output**: Product Delivered to the Customer

 **Abbreviations**: One Time Password (OTP), Controller (Pi), Return To Launch (RTL), Destination (DES), Amazon Web Service (AWS)

 *Initialisation*:

 Check Drone flight parameters

 Check Internet Connection

1: Get DES. Co-ordinates

 *LOOP Process*

2: **while** 1 **do**

3:  Take Off

4:  **if** *Altitude* >= *target* * 0.95 **then**

5:   *Return*

6:  **end if**

7:  *Wait_for*_1*sec*

8: **end while**

9: *goto*_*way*_*point*

10: **if** *OTP* == *Verified* **then**

11:  *Picture* ← *camera*.*pi*()

12:  *EdgeNode*(*Face* ← *detect*_[*picture*])

13: **else if** *Face* > = 1 **then**

14:  *CloudNode*(*Matching* ← *AWS*_[*picture*])

15: **else if** *Matching* == *True* **then**

16:  *deliver*_*product*

17:  *RTL*

18: **else**

19:  *RTL*

20: **else**

21:  *goto*_*Line*_11

22: **else**

23:  *RTL*

24: **else if**

### 4.4 Proposed Consumer-Auth algorithm

Algorithm 1, refers to the pseudo-code of the proposed Consumer-Auth framework. The proposed drone delivery mechanism consists of three primary inputs which include destination GPS co-ordinates, customer image, and customer mobile number. Moreover, the ultimate objective to achieve from the proposed system is to make it a reliable and secure drone delivery mechanism. After configuring the drone flight parameters and wireless connection, the proposed autonomous hub takes off its flight towards the customer’s destination. Furthermore, when the drone altitude is greater than 95% of its allocated target then it returns else it keeps going to attain that height. Further, after reaching the endpoint, it starts to continue the customer authentication process with the help of OTP and image recognition modules which ultimately terminate with product delivery. Despite this, if any module in the whole proposed system not properly execute then it calls the return to launch (RTL) process to keep it safe and secure.

## 5 Experimental setup

The experimental setup consists of three major components which include AWS cloud, Firebase, and drone that are used to implement the proposed Consumer-Auth framework. The AWS cloud and Firebase are the cloud services that are used to implement the proposed two-factor Consumer-Auth framework. On the other hand, the drone is our main hardware component that is used to pick a customer’s parcel from the service provider and drop the parcel at the destination after the two-factor verification of the recipient. We designed the drone skeleton and embedded four key modules on it. These modules include a camera, flight controller, edge node, and GPS controller. Table 2 shows the summarized specifications of the drone that we developed for a reliable drone-based delivery system. The flight controller manages the drone motors as well as its flight modes while the edge node performs computations and storage decentralization regarding the two-step consumer validation process with the assistance of cloud servers. The functional details of these components are given in previous sections 2 and 3.

**Table 2:** Drone specifications.

**Drone Type**	QuadCopter
**Drone Speed**	23.4Km/h
**Flight Mode**	Guided
**Maximum Distance Covered (m)**	X = 100, X = 150, X = 200, …, X = 500
**Battery**	12 V, 2600 mA
**Flight Controller**	Ardu Pilot Mega 2.8
**Edge Controller**	Raspberry Pi 4 (2GB)
**GPS Type**	NEO M8N
**Camera Type**	8 MP

**Fig 4:**
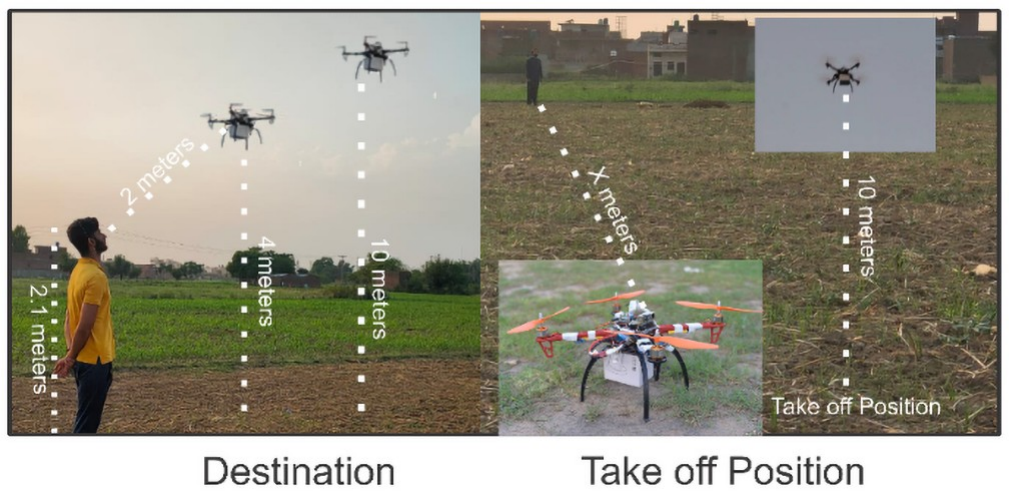
Experimental setup of the proposed Consumer-Auth framework.

The real-time testing of the proposed system is held under a non-constraint environment in an open field. Further, the experiment is conducted with various distances (X) from its initial point, i.e., 100m, 150m, 200m, …, 500m. As with 12V, 2600 mA battery bank, speed (v = 6.5 m/s), the maximum distance covered is 1Km (i.e., 500m one side distance) as shown in Fig 4.

### 5.1 Performance metrics

The proposed Consumer-Auth framework for a reliable drone-based delivery system, is evaluated on the basis of five common parameters which include distance(S), time taken (t), energy consumption (E), system accuracy (AC), and error rate (ER). These parameters are defined in the following subsections:

#### 5.1.1 Distance (S)

In order to calculate the circular distance for drone, we used Haversine theorem. The Haversine theorem calculates the distance on the basis of longitude-latitude initial and final co-ordinates along with the sin-cosine trigonometric identities. The mathematical relationship is given as Eq 1:

$$\begin{array}{c} \begin{array}{c} S1=sin^{2}(\frac{FLT-ILT}{2})\\ S2=sin^{2}(\frac{FLN-ILN}{2})\\ S=2*R*arcsin\sqrt{S1+cos(ILT)*cos(FLT).S2} \end{array} \end{array}$$ (1)

Here R = 6371.0 KM stipulates the radius of the earth, FLT refers to the latitude of ultimate location and ILT relates to latitude of the initial point whereas FLN refers to the final longitude and ILN is the initial longitude.

#### 5.1.2 Time (t)

The time (t) parameter is calculated on the basis of two factors, i.e., distance (S) and velocity (v). The mathematical representation is shown in Eq 2:

$$\begin{array}{c} t=\frac{S}{v} \end{array}$$ (2)

Here v represented fixed velocity which is 6.5m/s.

#### 5.1.3 Energy consumption (E)

Energy consumed by the proposed system is accounted by the dot product of current (I), voltage (V) and time taken (t). The mathematical representation is given in Eq 3:

$$\begin{array}{c} \begin{array}{c} E1=P*t\\ P=I*V\\ E=I*V*t \end{array} \end{array}$$ (3)

Here voltage (V) and current (I) are fixed i.e. 12V and 2600 mA.

#### 5.1.4 System accuracy (AC)

The system accuracy is defined as the percentage of correctly predicted observations with respect to all observations. Mathematically, it is given as Eq 4:

$$\begin{array}{c} AC=\frac{TP+TN}{TP+FN+TN+FP}\times 100 \end{array}$$ (4)

Here TP stands for true positive, TN stands for true negative, FP stands for false positive and FN stands for false negative.

#### 5.1.5 Latency overhead Hn (HLO)

It is the latency overhead factor that is calculated based on the total number of attempts for face detection and recognition. In the following equations, Eq 5 represents the latency overhead (LO) in a case when the face detection and recognition task is done at cloud (CLO) while Eq 6 represents the latency overhead when this task is done at edge device (ELO) only. Finally, Eq 6 represents the latency overhead when some of this task is done in at edge device and some at cloud level, i.e., in hybrid fashion (HLO).

$$\begin{array}{c} \begin{array}{c} CLO=n*Lc \end{array} \end{array}$$ (5)

$$\begin{array}{c} \begin{array}{c} ELO=n*Le \end{array} \end{array}$$ (6)

$$\begin{array}{c} \begin{array}{c} HLO=n*le+Lc \end{array} \end{array}$$ (7)

Here “n” is the total number of no face attempts, Lc = Cloud Latency (i.e., 10sec), Le = Edge Latency (i.e. 7sec) and le = detection time of edge node (i.e., 2sec)

## 6 Performance analysis

This section describes the experimental results of the proposed Consumer-Auth framework to the performance metrics as described in section 5.1.

### 6.1 Distance (S) and time (t) analysis

**Fig 5:**
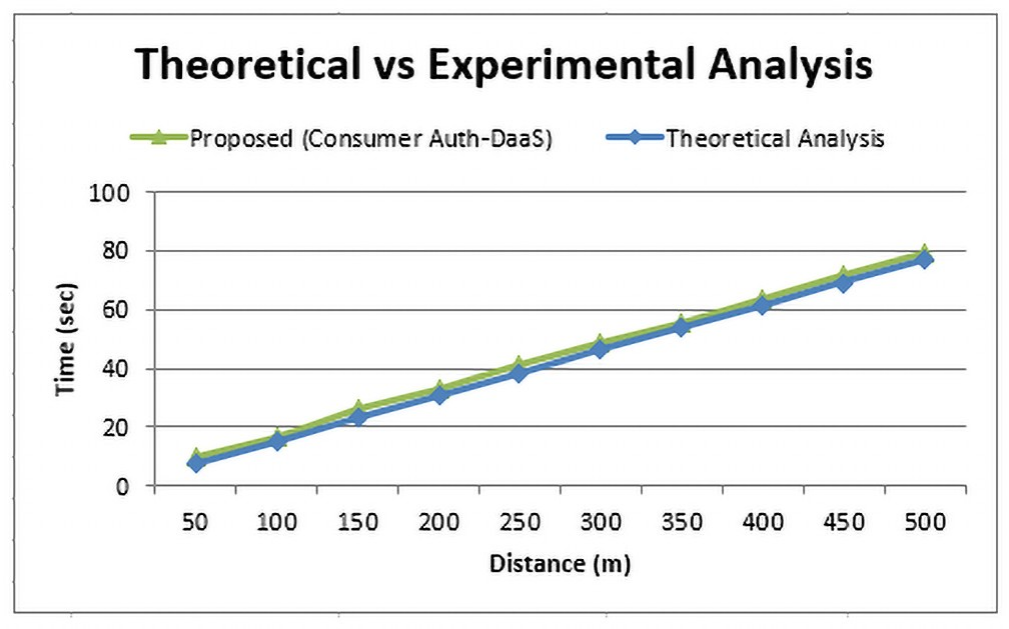
Time and distance relationship between the theoretical and experimental curves of the proposed Consumer-Auth framework.

Fig 5, illustrates the comparison between the total distance (in meters) traveled by drone and the overall time (in seconds) taken for both theoretical and real-time experiment perspective. In the theoretical analysis, we first calculated the expected time (t) by using Eq 2 and considering various distance points starting from 50m to 500m with an interval of 50m, i.e., 0m, 50m, …, 500m while keeping the velocity 6.5 ms. The theoretical, calculated time (t) parameter values are shown in Fig 5 with a blue line.

Based upon the theoretically calculated time (t) parameter values, we then noticed the drone GPS coordinates after theoretically calculated time value to calculate the distance (S) covered by the drone with the help of Harversine theorem as represented in Eq 1. We noticed a slighter difference between the theoretical and actual results. Besides, we also calculated the time taken by the drone for reaching the various distance points starting from 50m to 500m with an interval of 50m. And plotted the results in Fig 5 with a green line.

From Fig 5, it can be observed that there is a slight difference in both expected and actual time (t) parameter values for different distance points which show the efficiency of the proposed drone delivery mechanism. Moreover, this minimal difference is actually due to the lack of frictional parameters like air pressure, mechanical components, etc., in the theoretical equation which is obvious in the real-time environment. Furthermore, the distance for the elevation of the autonomous hub (drone) is completely dependent on the longitude and the latitude factor provided by the respective customer. As time is directly proportional to the distance, so if we increase the distance then the time parameter also increases accordingly. By keeping, in fact, the difference between the two curves, the value of distance in our first assessment was 50 meters, so the time factor concerning the theoretical module shows 7.7s but the actual time was recorded as 10.2s. Similarly, we vary the distance parameter on X-axis with the increment of 50 m and find the corresponding time to cover that distance as shown in Fig 5. Finally, by analyzing the output result of both test curves, it depicts the effectiveness of the proposed system.

### 6.2 Energy utilization (E)

**Fig 6:**
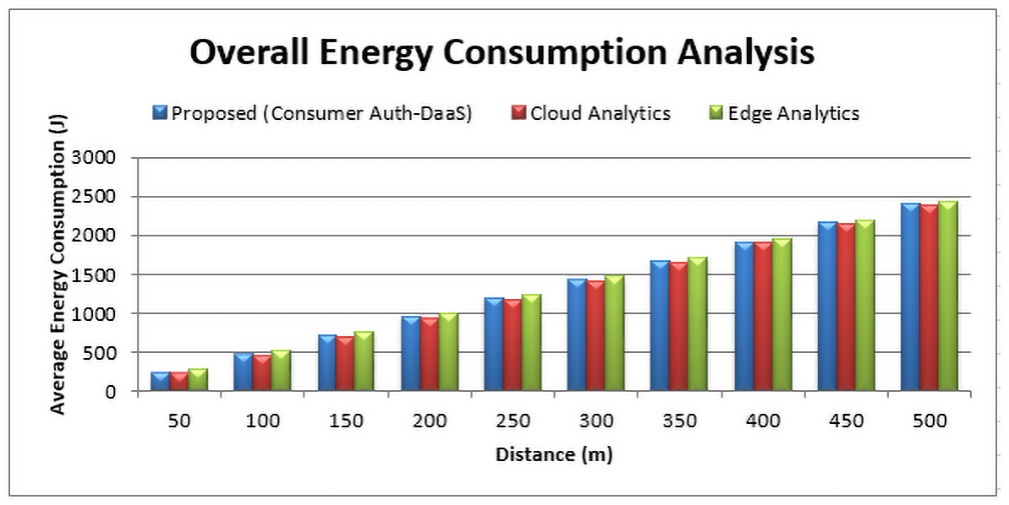
Energy utilization comparison among cloud computing, edge computing and the proposed hybrid computing based Consumer-Auth framework.

To evaluate the efficiency of the system, energy dissipation is one of the core factors. To calculate this parameter, we conducted multiple experiments for different distance marks as shown in Fig 6 through mathematical expression Eq 3. Moreover, we compared three core computing techniques, i.e., cloud, edge, and the proposed hybrid computing technique.

As refer to Fig 6, cloud computing outperforms in terms of energy consumption due to a remote computation platform as compared to edge computing. But, due to the latency overhead, we cannot prefer a cloud-based storage mechanism especially in drone delivery as a service (DaaS) where the latency factor is the crucial one. So, still, the cloud-based storage phenomenon utilizes less energy consumption, so we proposed a hybrid computing mechanism where both cloud and edge platforms are used. Similarly, in this way, we catered the latency overhead issue by utilizing a bit more energy than the cloud, but much less than edge computing as shown in Fig 6. By keeping in view the enormous benefits of the proposed Consumer-Auth framework using a hybrid computing concept, our system is highly efficient and robust than the existing techniques.

### 6.3 Face detection and recognition accuracy (AC)

**Fig 7:**
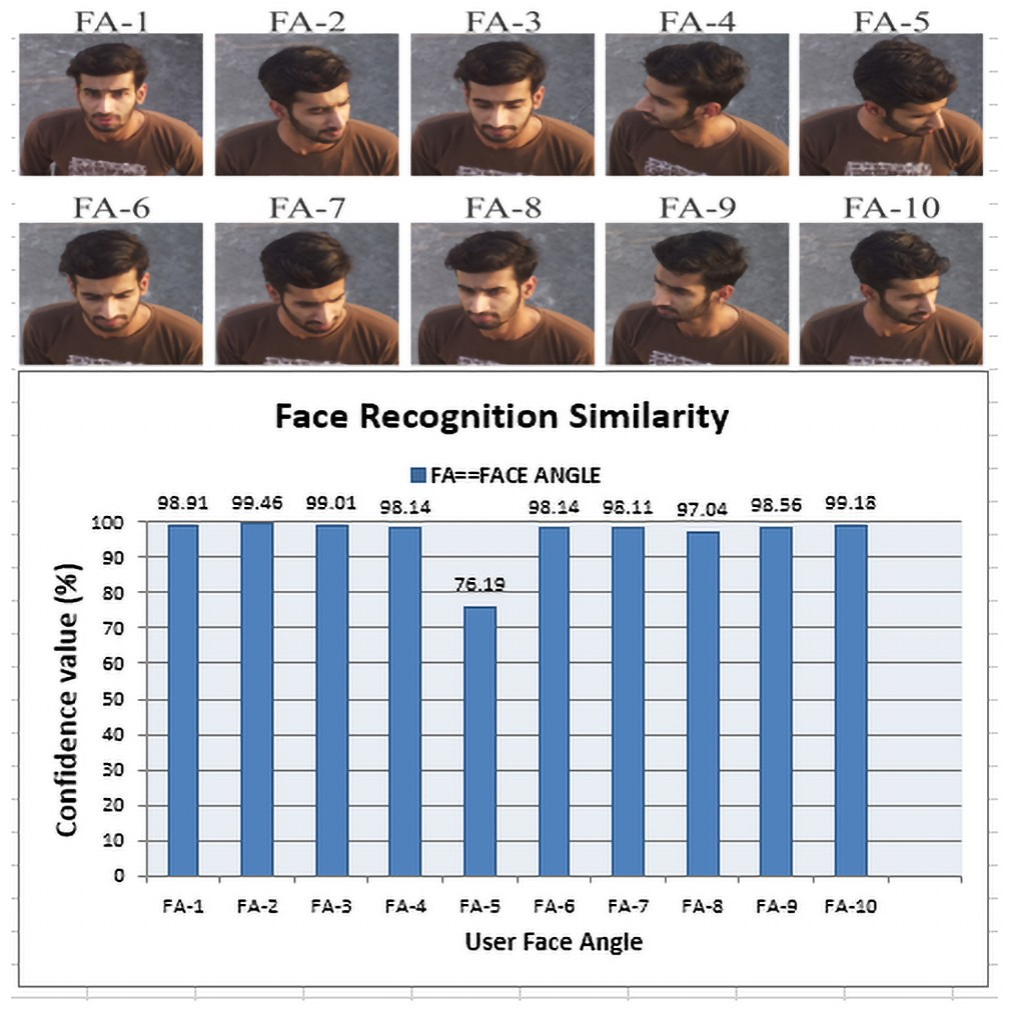
Face recognition accuracy with respect to different face angles (FA1—FA10) during the drone flight.

As previously discussed that we used the Haar algorithm for the detection of a face in an image captured by done. For face detection, we achieved 95% accuracy which is calculated using Eq 4. In Fig 7, it shows the face recognition percentage similarity in terms of confidence value which is computed on AWS cloud using the ‘AWS Rekognition’ module and mathematically expressed in Eq 4. It clearly shows the versatility of the AWS face recognition module even with different face orientations during an autonomous drone flight. To add more, in Fig 7, X-axis represents the face angles such as FA-1, …, FA-10, which are also visualized on the top of it as a specimen with appropriate labels. Besides, on Y-axis it shows the percentage confidence value also called a user face similarity rate. After analyzing the above values shown in Fig 7, the highest confidence value is achieved at FA-2, i.e., 99.46% while the lowest is achieved at FA-5 which is 76.19% respectively. Similarly, FA-3 and FA-10 are also recognized with > 99% confidence value, if we carefully observe FA-2 and FA-10, in both cases, it seems that the deep learning model is recognizing the person based on the side angle in which the nose edge feature is more prominent as compared to other facial features [30]. Based on the nose and other facial edge features, the ‘AWS Rekognition’ module recognized the person with more confidence interval in FA-2 as compared to FA-10. Likewise, if we compare the confidence interval of FA-1 and FA-10, due to more clear aerial view (canted angle) of nose for ‘AWS Rekognition’ module, the FA-10 has 0.27% more confidence value than FA-1. In short, even the face seems to be clear with the naked eyes, all facial features are not as prominent as in line-of-sight view. Therefore, the deep learning-based ‘AWS Rekognition’ module selects the most prominent facial features for processing through UAV. Hence, FA-10 is recognized with more confidence interval value as compared to FA-1.

In summary, we achieved 94.6% accuracy on average for face recognition. Even with a disoriented face angle as in FA-5, our proposed framework performs well to recognize a particular face. It depicts the authenticity of the proposed user authentication phenomenon in our Consumer-Auth framework. Finally, in Fig 7, we have shown all face angles for a single user as a specimen to analyze and evaluate the result appropriately.

### 6.4 Latency overhead (HLO)

**Fig 8:**
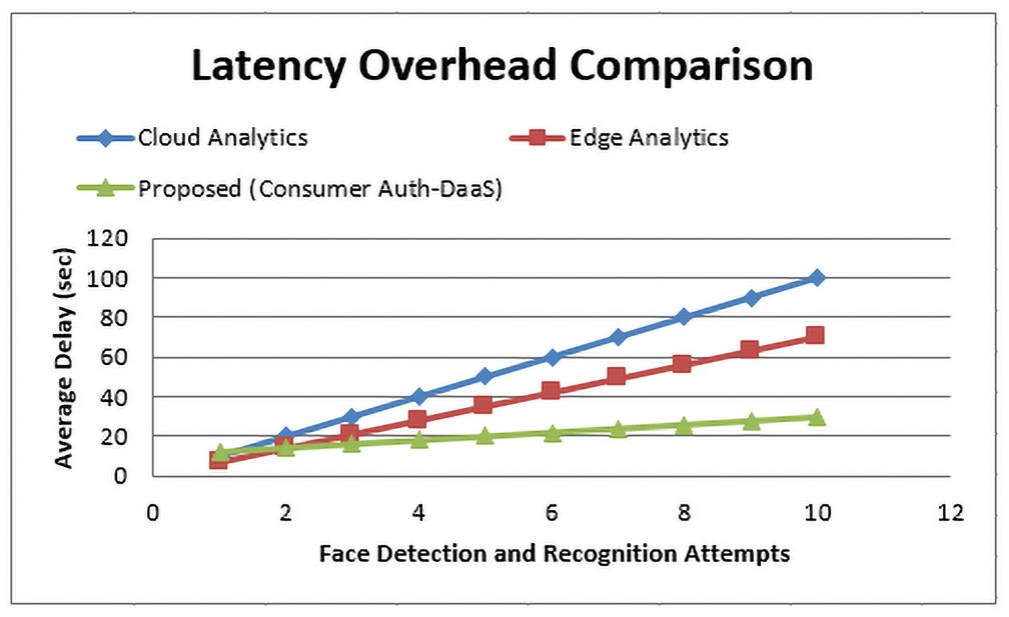
Latency overhead comparison among cloud computing, edge computing and the proposed hybrid computing based Consumer-Auth framework in terms of face recognition attempts.

Fig 8, depicts the latency overhead comparison for cloud, edge, and the proposed Consumer-Auth framework hybrid computing mechanism, mathematically represented by Eqs 5–7 respectively. We computed the latency overhead parameter when there is no face in the captured image from the autonomous drone. As we stated in sections 3 and 4, cloud computing platforms require an enormous response time to compute the image processing due to its remote physical existence. So, in a no-face scenario, the approximately double time required to compute another attempt for face recognition.

On the other hand, edge computing due to its processing constraints needs a lot of time to recognize a face in a case when multiple attempts are done. By keeping in view the above constraints of existing computing schemes, we can efficiently recognize the face on the AWS cloud only when the face detection is done at the edge node (as we did in the section 5.1.5, proposed Consumer-Auth framework. Fig 8 demonstrates as the number of attempts increases for face detection and recognition from 2 to 10, the cloud computing scheme shows the approximately linear response to the average delay, i.e., 5 sec to 100 sec. Whereas, in the edge computing phenomenon, the average delay is slightly less than the cloud-based approach, but still, it consumes a lot of time, i.e., 4 sec to 70 sec, to re-attempt the face detection recognition.

In contrast, the proposed Consumer-Auth framework performs phenomenally due to the adoption of the decentralized hybrid computing proposed system. However, the total number of face detection and recognition attempts didn’t put an adverse effect on the overall efficiency of the proposed system. The average overhead delay almost constant as the number of attempts increases from 2 to onward. As highlighted in Section 5.1.5, when the number of attempts is equal to “1”, all of the computing schemes requires approximately the same latency overhead delay. It didn’t affect the mathematical relationship due to its identity nature.

In this way, we can effectively utilize the AWS cloud face recognition service and also get rid of the latency penalty overhead in case of no face as shown in Fig 8. Finally, the proposed Consumer-Auth framework is highly responsive with minimal delay even if there is no face available in the captured image.

## 7 Conclusion

In this work, we proposed a consumer authentication (Consumer-Auth) framework to ensure a complete and reliable last-mile delivery through autonomous drones. Through the proposed Consumer-Auth framework, the drone first reaches the exact customer GPS location. Afterward, the drone authenticates the customer via a two-factor verification method which includes customer authentication via OTP and face recognition. Upon successful authentication of the intended customer, the drone delivers the ordered package otherwise it comes back without delivering the package. To ensure the timeliness, reliability, and robustness of consumer authentication, we proposed a hybrid computing model, i.e., cloud and edge computing architecture. Finally, we evaluated the proposed Consumer-Auth framework based on five common performance metrics as discussed in section 5.1. The proposed Consumer-Auth framework achieved a minimum confidence value of 76.19% and an average confidence value of 94.6%. The overall results demonstrate the effectiveness of the proposed Consumer-Auth framework to pledge a complete, reliable last-mile delivery via drones.

The performance of the proposed framework is limited to appropriate lighting and environmental conditions. However, in the future, we will consider the lighting and environmental parameters to improve the performance of the drone-based system. Furthermore, the operating range of the drone delivery system can be improved by using multi-hop based communication.
